# Application of mixed linear models for the estimation of functional effects on bovine stature based on SNP summary statistics from a whole-genome association study

**DOI:** 10.1186/s12711-022-00771-1

**Published:** 2022-12-16

**Authors:** Krzysztof Kotlarz, Barbara Kosinska-Selbi, Zexi Cai, Goutam Sahana, Joanna Szyda

**Affiliations:** 1 Biostatistics Group, Department of Genetics, The Wroclaw University of Environmental and Life Sciences, Kozuchowska 7, 51-631 Wroclaw, Poland; 2 Center for Quantitative Genetics and Genomics, Aarhus University, Blichers Allé 20, 8830 Tjele, Denmark; 3 National Research Institute of Animal Production, Krakowska 1, 32-083 Balice, Poland

## Abstract

Genome-wide association studies (GWAS) help identify polymorphic sites or genes linked to phenotypic variance, but a few identified genes and/or single nucleotide polymorphisms (SNPs) are unlikely to explain a large part of the phenotypic variability of complex traits. In this study, the focus was moved from single loci to functional units, expressed by the metabolic pathways as defined in the Kyoto Encyclopaedia of Genes and Genomes (KEGG) database. Consequently, the aim of this study was to estimate KEGG effects on stature in three Nordic dairy cattle breeds using SNP effects from GWAS as the dependent variable. The SNPs were annotated to genes, then the genes to KEGG pathways. The effects of KEGG pathways were estimated separately for each breed using a mixed linear model incorporating the similarity between pathways expressed by common genes. The KEGG pathway D-amino acid metabolism (map00473) was estimated to be significant for stature in two of the analysed breeds and revealed a borderline significance in the third breed. Thus, we demonstrate that the approach to statistical modelling of higher order functional effects on complex traits is useful, and provides evidence of the importance of D-amino acids for growth in cattle.

**Supplementary Information:**

The online version contains supplementary material available at 10.1186/s12711-022-00771-1.

## Background

Genome-wide association studies (GWAS) are very useful for the identification of polymorphic sites, typically single nucleotide polymorphisms (SNPs), or sometimes of genes associated with a phenotypic variation or with a disease. Today, the common availability of SNPs obtained based on whole-genome sequencing allows for a very good resolution of the estimation of those associations. However, in the context of phenotypes that show a complex mode of inheritance, a few genes and/or SNPs are not expected to be sufficient to explain the variability on a phenotypic level. As a consequence, in many cases, it is possible to identify loci with a very large effect on the phenotypic variation, but still, a predominant proportion of this variation remains unexplained [1], since it is often due to a combined effect of many loci, each with a moderate or small impact. Therefore, in our study, we moved the focus from a single locus to functional units, here expressed by the metabolic pathways defined by the Kyoto Encyclopaedia of Genes and Genomes (KEGG) database. This approach allows us to better understand the physiological mechanisms that underlie complex phenotypes. In the literature, the estimation of the effects of pathways has mainly been applied to gene expression data, but rarely in the GWAS context (see e.g. [2]). In this study, we used SNP summary statistics originating from the GWAS conducted for stature and based on whole-genome sequence data of three Nordic dairy cattle breeds. To our knowledge, this methodological approach proposes a new model, that is statistically most similar, albeit not equivalent, to the analysis described by [3].

## Methods

The analysed data comprised SNP summary statistics from GWAS performed on 5062 Danish Holstein bulls, 924 Danish Red Dairy Cattle bulls, and 2122 Finnish Red Dairy Cattle bulls [4]. The association was calculated for 25.4 million variants that were imputed with Minimac2 [5] from 630,000 SNPs using the 1000 Bull Genomes reference population from Run4, consisting of 1147 individuals. SNP additive effects were estimated for deregressed estimated breeding values (EBV) for stature. These were used as pseudophenotypes, separately for each breed, with a single SNP mixed linear model including an additive polygenic effect with a covariance described by a genomic relationship matrix. The model was implemented via the EMMAX software [6].

Based on their ID number, SNPs were annotated to genes corresponding to the ARS-UCD1.2 reference genome using the Bioconductor BioMart tool version 3.14 [7] and then genes were annotated to KEGG reference pathways (map) using the David software version 6.8 [8]. The effects of KEGG pathways on stature were estimated separately for each breed using the following mixed linear model that accounted for the similarity between pathways:

1 $$\mathbf{y}=\mathbf{1}\upmu +\mathbf{Z}\mathbf{t}+\mathbf{e},$$

where $\mathbf{y}$ is the vector of absolute values of SNP additive effects on stature that are estimated separately for each breed in the GWAS of Bouwman et al. [4], $\mathbf{1}$ is a vector of ones, $\upmu$ represents the general mean, $\mathbf{t}$ is the vector of the random effects of KEGG pathways with a preimposed normal distribution defined by $\mathrm{N}\left(0,{\mathbf{V}\upsigma }_{\mathrm{t}}^{2}\right)$, $\mathbf{e}$ is the vector of residuals distributed as $\mathrm{N}\left(0,{\mathbf{I}\upsigma }_{\mathrm{e}}^{2}\right)$, $\mathbf{Z}$ is the incidence matrix for $\mathbf{t}$. Note that if multiple SNPs were identified within a gene, only one SNP with the strongest effect was included in $\mathbf{y}$, so that each gene is represented by a single variant. The similarity between KEGG pathways $\mathrm{i}$ and $\mathrm{j}$, was introduced into the model by incorporating a non-diagonal KEGG covariance matrix $\mathbf{V}$. This covariance was expressed by the Jaccard similarity coefficient:

2 $$\mathrm{J}\left(\mathrm{i},\mathrm{j}\right)=\frac{\mathrm{M}}{\mathrm{N}},$$

where $\mathrm{M}$ represents the number of genes shared between KEGG pathways $\mathrm{i}$ and $\mathrm{j}$, while $\mathrm{N}$ represents the total number of genes involved in KEGG pathways $\mathrm{i}$ and $\mathrm{j}$. Variance components were assumed to be known: ${\upsigma }_{\mathrm{t}}^{2}=0.3{\upsigma }_{\mathrm{y}}^{2}$ and ${\upsigma }_{\mathrm{e}}^{2}=0.7{\upsigma }_{\mathrm{y}}^{2}.$

The mixed model equations [9] were used to obtain solutions for $\upmu$ and $\mathbf{t}$:

3 $$\left[\begin{array}{c}\widehat{{\varvec{\upmu}}}\\ \widehat{\mathbf{t}}\end{array}\right]={\left[\begin{array}{cc}{\mathbf{1}}^{\mathrm{T}}{\mathbf{R}}^{\mathbf{-1}}{\mathbf{1}}& {\mathbf{1}}^{\mathrm{T}}{\mathbf{R}}^{\mathbf{-1}}\mathbf{Z}\\ {\mathbf{Z}}^{\mathrm{T}}{\mathbf{R}}^{\mathbf{-1}}{\mathbf{1}}& {\mathbf{Z}}^{\mathrm{T}}{\mathbf{R}}^{\mathbf{-1}}\mathbf{Z}+{\mathbf{G}}^{\mathbf{-1}}\end{array}\right]}^{\mathbf{-1}}\left[\begin{array}{c}{\mathbf{1}}^{\mathrm{T}}{\mathbf{R}}^{\mathbf{-1}}\mathbf{y}\\ {\mathbf{Z}}^{\mathrm{T}}{\mathbf{R}}^{\mathbf{-1}}\mathbf{y}\end{array}\right],$$

where $$\mathbf{R}=\mathbf{I}{\widehat{\upsigma }}_{\mathrm{e}}^{2}$$ and $$\mathbf{G}=\mathbf{V}{\widehat{\upsigma }}_{\mathrm{t}}^{2}$$.

To maximise the computational performance of the estimation/prediction process, a custom Python program implementing the NumPy 1.19.5 library [10] was used. Since all calculations were carried out on a high-performance server, the NumPy library was also used to set the array indexing and order, which further improved the computing time compared to a native Python application. Each element of $\widehat{\mathbf{t}}$ was assessed for significance (${\mathrm{H}}_{0}:{\widehat{\mathrm{t}}}_{\mathrm{i}}\le 0$ vs. ${\mathrm{H}}_{1}:{\widehat{\mathrm{t}}}_{\mathrm{i}}>0$) by calculating the probability of obtaining a more extreme value from the $\mathrm{N}\left(0,{\upsigma }_{\mathrm{t}}^{2}\right)$ density function. Since NumPy and SciPy application programming interfaces (API) are implemented with LAPACK and BLAS, which require Fortran memory layout, all input matrices were transformed to Fortran to avoid costly transposing. In comparison to a fixed matrix input, this approach results in a ten times faster estimation process.

## Results and discussion

**Fig. 1:**
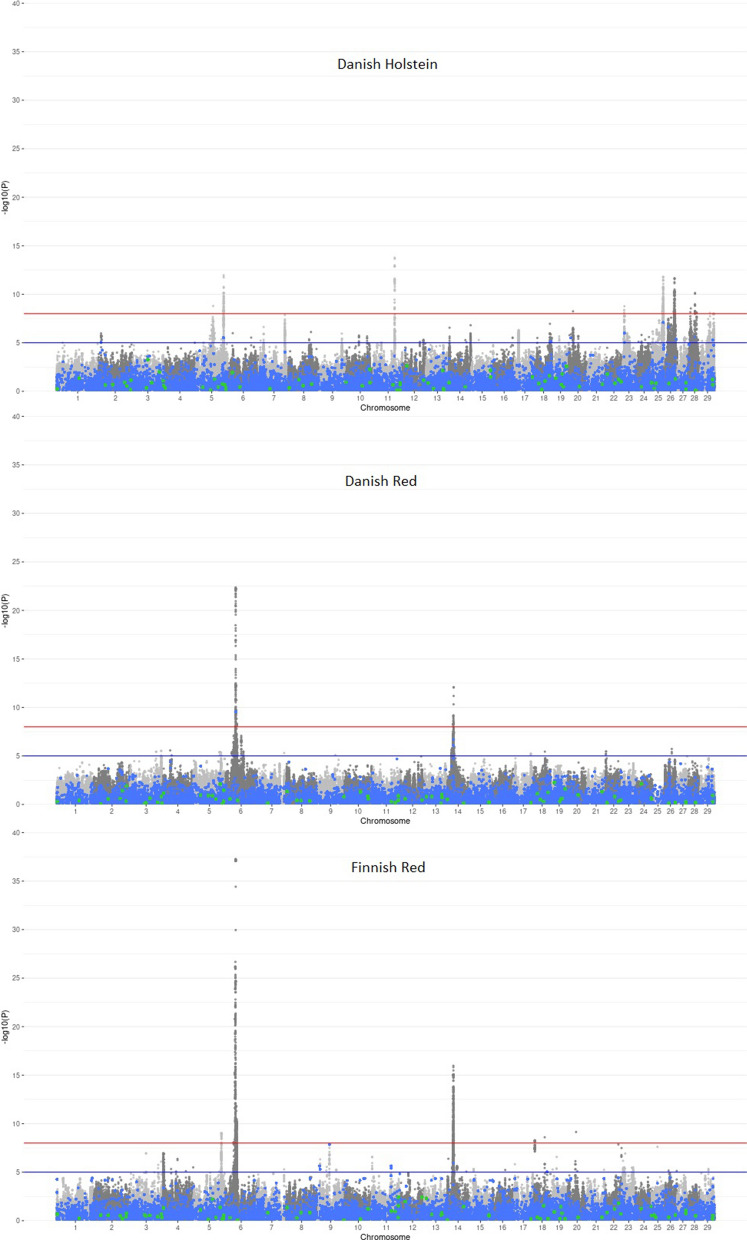
SNP significance from the whole-genome sequencing study of Bouwman et al. [2]. Blue dots correspond to SNPs in genes used for the estimation of the effects of KEGG pathways in model (1), green dots correspond to SNPs marking genes constituting the map00473 pathway, and grey SNPs are the remainder

**Fig. 2:**
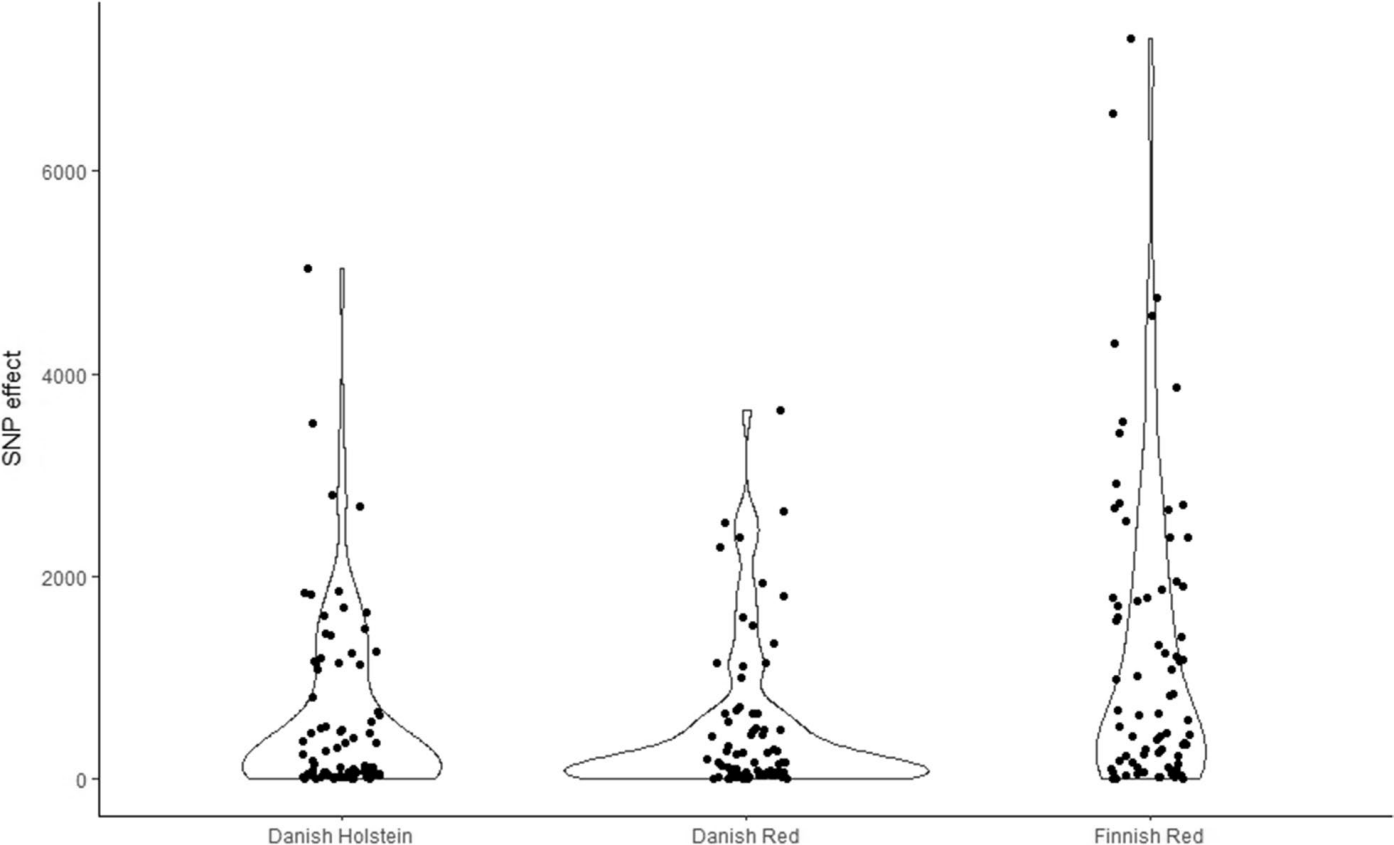
Estimated effects of SNPs marking genes from the map00473 pathway

The effects of 179 KEGG pathways were estimated based on the effects of selected SNPs from a whole-genome sequence-based GWAS of Bouwman et al. [4], separately for three Nordic cattle breeds—Danish Holstein (DH with 366,877 SNPs), Danish Red Dairy Cattle (DR with 299,723 SNPs), and Finnish Red Dairy Cattle (FR with 396,224 SNPs) (Fig. 1). In DH and FR, the same pathway—D-amino acid metabolism (map00473) revealed a significant effect on stature with moderate P-values of 0.035 (FR) and 0.049 (DH). In DR, it also reached a borderline significance of 0.133. Depending on the breed, the effect of map00473 was estimated based on 78 SNPs in DH and FR, and 76 SNPs in DR (Fig. 2 and Additional file 1: Tables S1 and S2). The differences in SNP counts resulted from the fact that the input SNP panel in Bouwman et al. [4] was pre-processed separately for each breed, which resulted in breed-specific SNP exclusion. In addition, the pathway responsible for the metabolism of terpenoids and polyketides (map01059) was significant (P = 0.041) in DH, while the synthesis and degradation of ketone bodies pathway (map00072) and the pathway of biosynthesis of various plant secondary metabolites (map00999) were significant in DR with P = 0.037 and P = 0.047, respectively.

The idea to incorporate pathway effects directly into a mixed GWAS-based model of complex phenotypes dates back to 2014 and was presented by Evangelou et al. [11] who proposed two models and corresponding estimators of their effects. A difference between the models presented in [11] and our model is the use of measured phenotypes as a dependent variable in their case vs SNP summary statistics as a dependent variable in our case. Moreover, Evangelou et al. [11] defined no covariance between pathways, but allowed pathway-specific variances, while in our model we used a non-diagonal covariance.

When interpreting the effects of KEGG pathways, two scenarios emerge. On the one hand, the overall strong effect of a pathway may be driven by the strong effect of a single gene, that is this pathway’s component—a situation that could have been detected in a conventional GWAS. On the other hand, the strong effect of a pathway may be due to the combined effects of many genes constituting this pathway—a situation that may easily be missed in GWAS due to the small or moderate effects of particular genes from the pathway. In the case of our data—none of the genes that harbour the most significant SNPs in the GWAS performed by Bouwman et al. [4] were a component of the D-amino acid metabolism pathway, which therefore leads to the conclusion that the whole pathway is a significant component of the genetic determination of stature. Biologically, an outstanding pattern of our study was that the pathway associated with the metabolism of D-amino acids is significant for two breeds and on the border of claimed significance in the third breed. Although D-amino acids do not occur in naturally-translated proteins, the link between D-amino acids metabolism and growth has long been recognised. Experimentally, a supplementation of mice with D-amino acids resulted in increased weight that was observed with an increased concentration of D-phenylalanine and D-tryptophan in the diet [12]. Moreover, D'Aniello [13] reported that, in the pituitary gland, D-aspartic acid stimulates the secretion of the growth hormone in rats. In cattle, a supplementation of food with synthetic amino acids is a very common practice with commercial diet supplements containing a mixture of naturally occurring L-versions as well as not naturally occurring D-versions. Campbell et al. [14] observed that D-amino acids are somewhat less efficiently metabolised than their naturally occurring synonyms. Since methionine is often the first limiting amino acid for growth in cattle [15], individuals that possess a more efficient mechanism of D-amino acid metabolism are expected to grow better, which may result in higher stature in adults.

Another metabolic pathway demonstrating potential importance on stature is the synthesis and degradation of the ketone bodies pathway (map00072) that was significant in DR. It has been demonstrated that the metabolism of ketone bodies is related to the growth of the whole organism (mainly through the *SLC16A6* gene as reported by Kichaev et al. [16] and Karanth et al. [17]) and also that it acts at a single-cell level by triggering mitochondrial response towards cell’s oxidative stress and deficiency in metabolic energy [18]. Although the other pathway of biosynthesis of various plant secondary metabolites that was significant in DR, does not relate directly to animal metabolism, it can be hypothesised that genes playing a role in the biochemical processing of metabolites originating from plants lead to higher feed efficiency in cattle and furthermore influence animals’ growth, but experimental evidence is lacking.

## Conclusions

Our results demonstrate that taking higher-order components of biological systems, such as metabolic pathways, into consideration, provides valuable insight into the basis of the variation of complex phenotypes, that may be missed by conventional GWAS and should be used as an enhancement thereof. From a practical perspective, it would imply conducting genomic selection not only on the most significant, major genes but also considering “additional” genes that are members of the significant metabolic pathways. This shall be especially useful for phenotypes that have undergone unidirectional selection for several decades, such as milk production traits, for which it can be expected that most of the major genes are already close to being homozygous for causal mutations.

## Supplementary Information

**Additional file 1: Table S1.** Gene effects in map00473. Estimated gene effects (represented by the most significant SNP) constituting the map00473 KEGG pathway. **Table S2.** KEGG pathway effects. Estimated effects of all KEGG pathways. Significant pathways (P ≤ 0.05) are marked in blue.

## Data Availability

BioSample accession codes to raw data are provided in https://doi.org/10.1038/s41588-018-0056-5.
